# Surface Treatment by Physical Irradiation for Antifouling, Chlorine-Resistant RO Membranes

**DOI:** 10.3390/membranes13020227

**Published:** 2023-02-13

**Authors:** Marwa S. Shalaby, Heba Abdallah, Ralph Wilken, Schmüser Christoph, Ahmed M. Shaban

**Affiliations:** 1 Chemical Engineering Department, Engineering Research & Renewable Energy Institute, National Research Centre, 33-El Buhouth Street, Dokki, Cairo 12622, Egypt; 2 Plasma Technology and Surface Treatment Department, Fraunhofer Institute for Manufacturing Technologies and Advanced Materials (IFAM), Wiener Straße 12, 28359 Bremen, Germany; 3 Water Pollution Research Department, Environmental Research Institute, National Research Centre, 33-El Buhouth Street, Dokki, Cairo 12622, Egypt

**Keywords:** vacuum UV, plasma treatment, thin-film composite, chlorine resistance, fouling behavior

## Abstract

Reverse osmosis (RO) membranes represent a strategic tool for the development of desalination and water treatment processes. Today’s global needs for clean water supplies show stressing circumstances to secure this supply, relying upon desalination and wastewater treatment and reuse, especially in Egypt and the Middle East. However, chlorine attack and fouling of polyamide layers, the active (selective) layers of RO membranes, are representing a great obstacle to seriously spreading the use of this technology. One promising way of fouling control and chlorine resistance is surface modification using grafting by plasma or vacuum ultraviolet (VUV) irradiation as a layer-by-layer assembly on polyamide membranes. Several studies have shown the effect of grafting by plasma using methacrylic acid (atmospheric pressure plasma) and showed that grafted coatings can improve PA membranes toward permeation compared with commercial ones with fouling behavior but not chlorine resistance. In this work, the techniques of layer-by-layer (LBL) assembly for previously prepared PA RO membranes (3T) using a mixed-base polymer of polysulfone and polyacrylonitrile in the presence of nanographene oxide (GO) without chemical grafting and with chemically grafted poly-methacrylic acid (3TG) were used. Membranes 3T, 3TG, a blank one (a base polymer membrane only was surface modified using VUV activation (AKT), and one with a grafted layer with polyethylene glycol (VUV-PEG) were prepared. These were then compared with polydimethylsiloxane (VUV-PDMS) and another surface modification with low-pressure plasma using acrylic acid (acryl) and hexadimethyl siloxane (GrowPLAS). The tested membranes were evaluated by short-term permeation and salt rejection experiments together with fouling behavior and chlorine resistance. A clear improvement of chlorine resistance and antifouling was observed for 3T membranes under plasma treatment, especially with the grafting with polyacrylic acid. Better antifouling and antichlorine behaviors were achieved with the vacuum UV treatment.

## 1. Introduction

RO membranes have been playing an important role in securing freshwater resources through wide applications in water desalination. RO technology is governed by high performance for such membranes, and nowadays, the widely used commercial polyamide RO membranes are known for their high salt rejection rates and moderate water flux [1,2]. However, this classical polyamide layer always suffers from chlorination attacks and fouling [3], which deteriorate the separation performances of RO membranes and shorten their lifetimes. This affects their spread into wide applications, especially in lower-income countries, such as Egypt. The preparation of antifouling, chlorine-resistant RO membranes is considered an effective way to extend membrane lifetime using cost-effective modifications, such as layer-by-layer deposition to protect the polyamide layer. 

Surface modifications can be considered an effective remedy for RO membranes either chemically or physically. In this article, physical treatment for membrane surfaces will be modified by plasma and vacuum irradiation (VUV) to increase hydrophilicity and biocompatibility, leading to antifouling [4]. Membranes are either surface activated or irradiated with a monomer or any other vapor or solution additive. The irradiation could be atmospheric plasma, VUV irradiation, low-temperature plasma, X-ray irradiation, or others [5]. Furthermore, VUV irradiation is a very useful technique for its low-temperature, low-investment-cost, simple, and rapid operation. Modification via VUV produces a top layer on the membrane surface with high selectivity due to the strong chemical bond to the substrate. This will lead to a more mechanically stable membrane, especially in operation at high pressures, and protects the mechanical properties of the base polymer. The irradiation does not modify the top layer only but the base polymer too [6,7]. Selected polymers treated with VUV radiation are oxidized when exposed to air with helium/argon. Reactions at the membrane surface may change with the exposure time and oxygen/gas flow rates introduced into the vacuum system [8]. 

During plasma surface modification, the surface is bombarded by plasma ions. The formed reactive species of the gaseous precursor interact with the surface and are incorporated into the outermost surface of the membrane [9]. Peroxides and hydro-peroxide are created on the surface of the sample when exposed to air after plasma irradiation. These groups will act as initiators for grafting polymerization. Membrane samples treated with low-pressure plasma use gaseous monomers in the presence of oxygen and argon gas with variable exposure times governed by adhesion and wettability [10,11]. However, surface grafting can be fulfilled via a redox reaction [12,13], but it becomes more stable with atmospheric or low-pressure-plasma treatment [9] together with ultra-violet (UV) irradiation [14,15]. There is little published work for surface grafting using VUV treatment or / plasma irradiation for RO membranes. Previously, Tirado and his team have grafted poly 2-[(methacryloyloxy) ethyl] dimethyl-(3-sulfopropyl) ammonium hydroxide (PSPE) to the surface of polyamide-RO membranes using ultraviolet. The hydrophilicity of the modified membrane was increased with better resistance to bacterial adhesion. The surface modification of the RO membrane was carried out by grafting per-fluorophenylazide terminated PEG (PFPA-PEG) using ultraviolet light [16]. The pure water permeability of the PEPA-PEG-grafted membrane showed a decline, but hydrophilicity, salt rejection, and fouling resistance were enhanced [17]. Many researchers explained that the improved surface hydrophilicity was induced by forming new oxygen-containing groups on the membrane surface, such as –OH, –OOH, etc., and others that are hydrophilic [18]. In this work, the authors will show the effect of plasma and vacuum ultraviolet irradiation on different TFC RO membranes. These membranes were prepared with polysulfone and polyacrylonitrile blends as the base polymers and were cast over nonwoven polyester. Plasmapoylmeric coatings and VUV-cured coatings will be presented using different monomers to form polyacrylic acid, poly-dimethyl siloxane, and polyethylene glycol. Surface modifications with/without activation will be examined along with their influence on permeation, salt rejection, chlorine resistance, and antifouling behavior.

## 2. Materials and Methods

A4-Membrane samples were prepared using a blend base polymer of polysulfone and polyacrylonitrile in the presence of graphene oxide nanoparticles as described briefly in [19]. This formulation was one of five different formulations regarding additives which were used in the polymeric dope solution and was found to be the optimum one and nominated “3rd family”. These membranes were prepared by dissolving 19% polysulfone, 1% polyacrylonitrile, and 0.25% nanographene oxide in N-N, dimethylacetamide (DMAc) for 24 h to form a homogeneous dope solution. The polymeric dope solution was cast on nonwoven polyester support, and the membranes were fabricated by non-solvent-induced phase separation (NIPS). The nominated 3 (blank membrane) was used to prepare a polyamide layer by interfacial polymerization of meta phenylene diamine and trimezoyl chloride and nominated 3T. Again, the blank membrane was coated with a hydrophilic, crosslinked layer of polyvinyl alcohol PVA-Glutaraldehyde GA and nominated 3P. A chemical grafted layer of poly-methacrylic acid/graphene oxide was formed for both 3T and 3P to form 3TG and 3PG, respectively.

These membranes were subjected to irradiation for surface modification by means of VUV and low-pressure plasma (Table 1) for membrane surface activation and grafting to present their effect on the performance of RO membranes in terms of brackish water desalination, chlorine resistance, and antifouling behavior. Membrane performance was measured by flat-sheet membrane testing cell with a constant feed pressure of 20 bar.

### 2.1. Vacuum UV (VUV) Treatment and Low-Pressure Plasma

**Figure 1:**
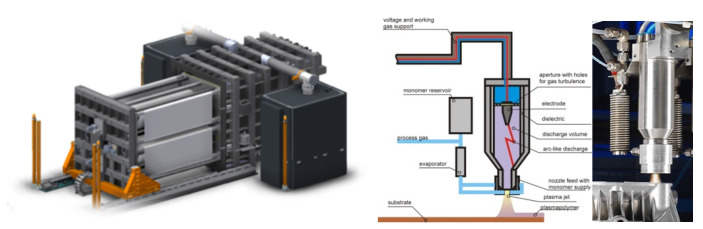
Plasma treatment systems: low-pressure plasma system (**left**) and atmospheric pressure plasma (**middle**) and (**right**).

Atmospheric plasma surface treatment is considered ideal for in-line processes in addition to robot-assisted guidance. The treatment of small areas, tracks, and large areas is possible too due to presence of a movable treatment system with the ability to activate polymeric surfaces and promote adhesion by functionalization, as shown in Figure 1. Additionally, low-pressure plasma systems can treat complex shaped 3-dimensional parts but also web materials, such as polymeric films and membranes.

**Figure 2:**
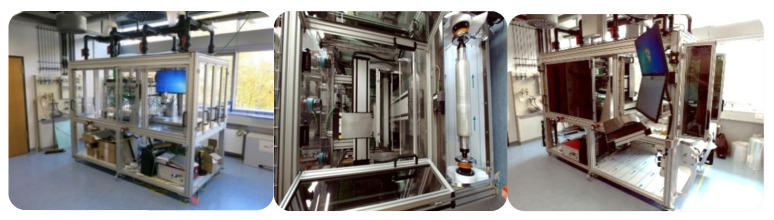
VUV setup.

Compared to plasma treatment, the activation step allowed the polymer adhesion to be performed by VUV irradiation with more advantages, as it can modify not only the outermost surface of the polymeric film but also the subsurface. This effect could result in optimized long-term durable functions, such as wettability or reactivity. The used VUV setup is depicted in Figure 2.

The treatments are described in detail as follows:

1. VUV Activation (AKT)

A4 sheets of different membranes were subjected to vacuum ultraviolet (VUV) using a traversing table and different kinds of VUV modules without prior cleaning in an ultrasonic bath (Bandelin Sonorex RK103H) with 1 min as exposure time in deionized water. In case of Xe-excimer radiation (emission wavelength 172 nm), a combination of seven lamps (Radium: XERADEX L40/620/DB-AZ48/90 OG) was used.

2. VUV-PEG

Polyethylene glycol (PEG) was dissolved in water. The solution was atomized to form a PEG-containing aerosol. This aerosol was directed onto the membrane surface to form a uniform thin layer of PEG. In a second step, the PEG was crosslinked and cured by the abovementioned VUV irradiation process.

3. VUV-PDMS

Poly-dimethylsiloxane is a higher-molecular-weight silicone oil that was diluted with hexamethyl di-siloxane so that it could be applied to the membrane by aerosol. A thin layer was deposited on the membranes, which had a thickness of approx. 30 nm after VUV crosslinking.

4. Low-pressure plasma (acryl-plasma)

Acrylic-based plasma polymer (polyacrylic acid): The deposition of the plasma polymeric coating was performed using a plasma reactor equipped with an RF source (13.56 MHz). After a short surface activation using oxygen, the vapor of acrylic acid was fed into the plasma zone and was activated and fragmented in the plasma zone. Fragments of the acrylic acid condensated on the substrate surface (membrane) and formed a plasma polymeric coating. The plasma polymerization used argon and acrylic acid vapor as a precursor. The pressure in the vacuum chamber was 7.5 × 10^−3^ mbar at a power of 30 W and a deposition time of 3 min; the resulting coating had a thickness of 30 nm. The cleaning of the substrate material was performed with pure argon at 30 W power for 60 s. The acrylic acid coating was found to have hygroscopic properties.

5. Low-pressure Plasma (GrowPLAS)

In the case of the low-pressure plasma coating, GrowPLAS hexamethyl disiloxane was fed as a precursor into a low-pressure plasma chamber. The hexamethyl disiloxane fragments were formed with the same abovementioned technique in acryl plasma and deposited as a very thin layer (25 nm) on the surface. The process parameters were chosen to produce a hydrophilic layer. The chemically treated membrane samples were surface irradiated (see Table 1).

### 2.2. Membrane Performance

Performance evaluation of surface-modified membranes was investigated. The permeation tests were carried out in a crossflow, flat-sheet testing cell for brackish water desalination unit at ambient temperature and under operating pressure up to 25 bar; the feed solution tank had capacity of 20 L. The effective surface membrane area was (54 cm^2^). Synthetic feed solution was prepared using sodium chloride (1 g/L) and calcium chloride (0.2 g/L) with total salinity of 1000–1050 ppm. Experiments were operated at constant pressure of 20 bar. Membrane samples underwent permeation test for 4 h using distilled water before testing.

The total permeate flux (J) of the tested solution was determined from Equation (1):

J = (Q /A × t)(1)

where Q is the permeate mass in kg; A is membrane active area in m^2^; and t is time in hour

The rejection (*R*) was calculated as Equation (2):

(2) $$R\%=\frac{(C_{f}-C_{P})\times 100}{C_{f}}$$

where *C_f_* is the concentration of the salt-feed solution, and *C_p_* is the concentration of salt in permeate.

### 2.3. Membrane Fouling Testing

Experiments were carried out using crossflow, flat-sheet lab unit described in the above section for membrane fouling behavior by using mixed foulant model which was composed of a polysaccharide (sodium alginate (2 g/L) (SA)) and protein (bovine serum (1 g/L) (BSA)) and added and mixed thoroughly with sodium chloride (1 g/L) and calcium chloride (0.2 g/L). Firstly, the synthetic salt water (1 g/L NaCl and 0.2 g/L CaCl_2_) was permeated through the membranes for 100 min, and the permeate flux (LMH) Jw1 was measured. Secondly, the mixed foulant solution was permeated through membranes for another 200 min. The permeate flux Jp (LMH) was measured based on the amount of produced water permeated from the membranes.

The fouled membranes were rinsed with distilled water to remove remains of fouling solution. Finally, synthetic salt water was passed through membranes again for 100 min, and the permeate flux was measured Jw2 (LMH). The flux recovery ratio (FRR) was calculated as follows (Equation (3)):

(3) $$\mathrm{FRR}\%=\frac{\;\mathrm{Jw}2\;}{\mathrm{Jw}1}\times 100$$

The total fouling ratio (Rt) was calculated as shown in Equation (4):

(4) $$\mathrm{Rt}\;\%=\left(1-\frac{\mathrm{Jp}}{\;\mathrm{Jw}1}\right)\times 100$$

where Rt is the degree of total flux loss caused by total fouling. Reversible fouling ratio (Rr) and irreversible fouling ratio (Rir) can be calculated by following equations, respectively, Equations (5) and (6):

(5) $$\mathrm{Rr}\%=\frac{\left(\;\mathrm{Jw}2-\;\mathrm{Jp}\right)\;}{\mathrm{Jw}1\;}\times 100$$

(6) $$\mathrm{Rir}\%=\frac{\left(\;\mathrm{Jw}1-\;\mathrm{Jw}2\right)\;}{\mathrm{Jw}1\;}\times 100$$

Generally, Rt is the sum of *R*r and *R*ir.

### 2.4. Membrane Chlorine Resistance

Sodium hypochlorite solution was freshly prepared by dissolving 13% by weight in distilled water corresponding to 1 g/L (1000 ppm) as free chlorine. This solution was taken as high dose to observe changes in membrane permeation and salt rejection after soaking for 24 h. The evaluation was performed by calculating the separation percentage and water permeation using crossflow, flat-sheet lab testing unit for two different samples of the same membrane. The average values are presented below.

### 2.5. Analytical Techniques

1. X-ray Photoelectron Spectroscopy

The XPS spectra were carried out on a VG ESCALAB 220i-XL spectrometer with a monochromatic Al Kα (1486.6 eV) X-ray source and operated at 15 kV and 20 mA. The base pressure of the chamber was below 4 × 10^−8^ Pa. The instrument was equipped with a magnetic lens system to increase the yield of detected photoelectrons. Spectra were acquired with a takeoff angle of 0°. Two independent measurements were performed at each sample, and the results were averaged.

2. Contact angle

Contact angle measurements are used to characterize the hydrophilic property of polymeric surfaces. The contact angle of membranes was measured using a compact video microscope (CVM) manufactured by SDL-UK; the contact time was 10 s with average drop volume of 10 μL, and each value was averaged from 10 measurements.

3. Scanning Electron Microscope (SEM)

It was used to observe the morphology of prepared membranes and surface-modified ones whereas the samples for cross-sectional view were coated with gold to provide electrical conductivity. The cross-sectional and top surface snapshots of membranes were taken with a JEOL 5410 scanning electron microscope (SEM) and conducted at 10 kV.

## 3. Results and Discussions

### 3.1. Effect of Different Physical Irradiations on RO Membranes-3T

Different irradiation was applied to different top-layer membranes, and their influence was recorded as permeate flux and salt rejection. In this section, 3T (TFC only) was subjected to the previously mentioned VUV and plasma irradiation.

Two plasma surface modifications were formed: one through GrowPLAS, which is nominated GrowPLAS, and the other through poly acrylic acid grafting by means of plasma, nominated Acryl. Vacuum UV was equipped with three different techniques: activation (AKT), surface functionalization by polyethylene glycol (PEG)-VUV-PEG, and PolyDiMethylSiloxane (PDMS) VUV-PDMS. The main challenge in this evaluation for RO membranes was the compromise between increased flux and salt rejection percent with chlorine-resistant antifouling behavior.

**Figure 3:**
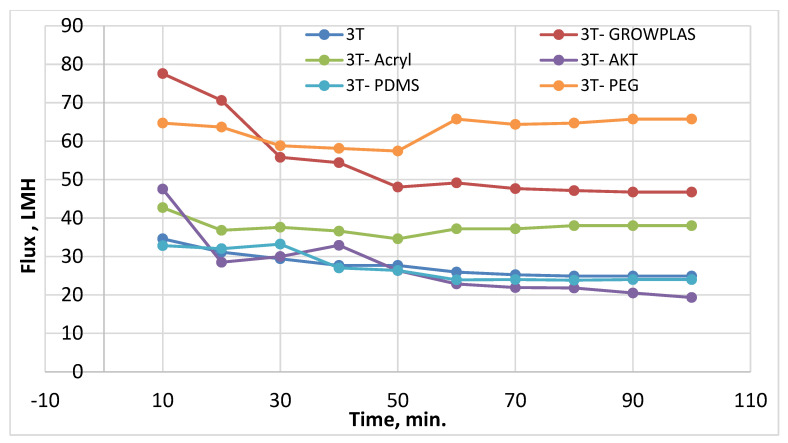
3T Flux variation with permeation time for different irradiations.

**Figure 4:**
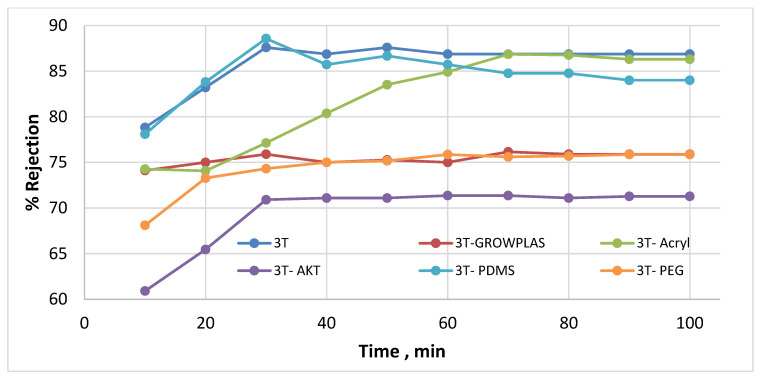
3T % rejection variation with permeation time for different irradiations.

**Figure 5:**
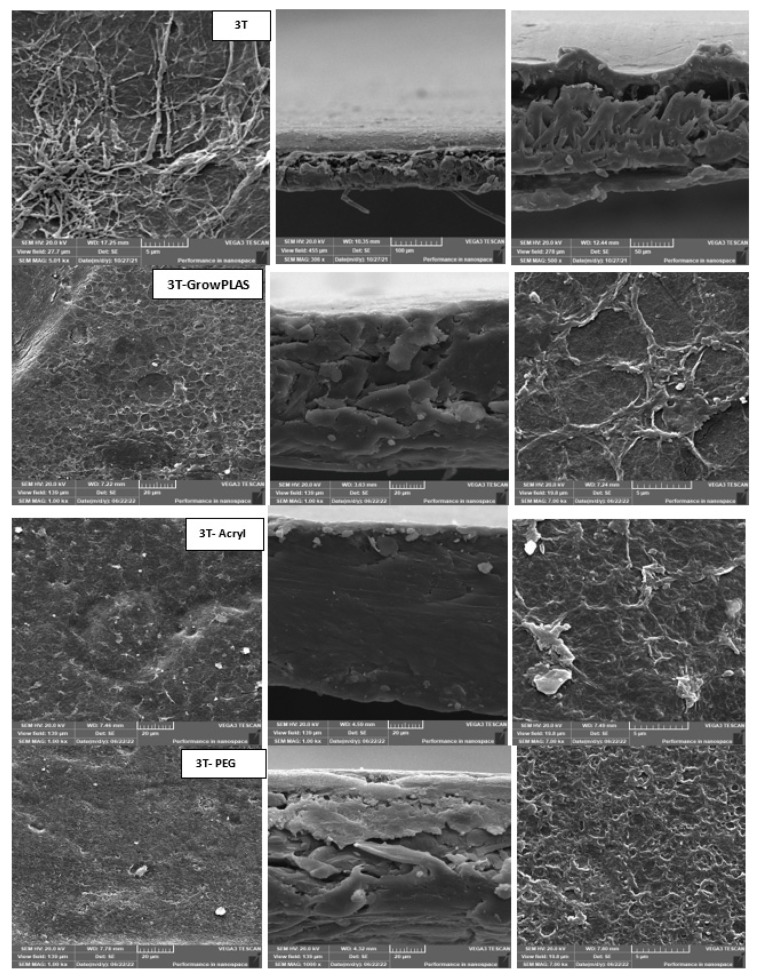

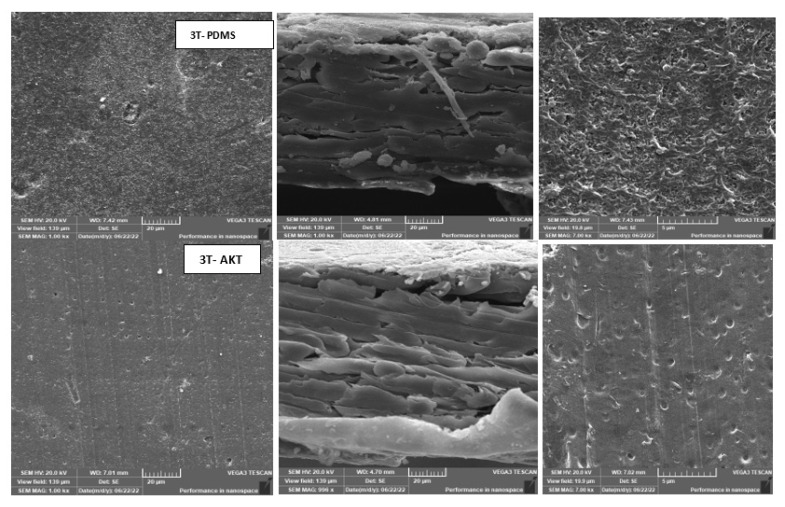
3T SEM morphology for top surface and cross-section for different treatments.

As shown in Figure 3 and Figure 4, the maximum percentage rejection was about 86% for 3T (polyamide only) without surface irradiation, which is the baseline of evaluation. It seems that certain pores are slightly affected by plasma treatment, and grafting with polyacrylic acid salt rejection increased from 74% after 10 min of operation and stabilized at 86%. This corresponds to about 42 LMH permeated water flux, which stabilizes at 37 LMH and is better than 3T permeation, which shows lower values from 32 to 26 LMH. However, the behavior of vacuum UV with grafting of polydimethylsiloxane (VUV-PDMS) showed the same membrane permeation of water with a slight decrease in salt rejection to 84%. In Figure 5, one can easily notice the formation of the dense layer in the cross-sectional plot for 3T-acryl and 3T PDMS, resembling that in 3T, and this shows a better interpretation of the reached values in Figure 3 and Figure 4. This is completely different in 3T-GrowPLAS and 3T-AKT, where open pores can be distinguished easily in the SEM morphology.

The plasma irradiation using GrowPLAS shows a destructive action accompanied by the presence of open holes and deformations in Figure 5, which increased permeate flux to about 77 LMH with a quick decline in rejection percentage to 76% when compared to 3T without irradiation, as shown in Figure 3 and Figure 4. The same can be concluded for VUV-PEG for lowering the rejection percent from 87 to 75% and the deformations in the TFC layer. The best surface modification for 3T was noticed after coating 3T with a plasma polymeric layer of polyacrylic acid (acryl), which represents an increase in the dense structure of cross-sectional morphology (Figure 5). Moreover, a sensible increase in permeate flux reached 38 LMH (acryl) when compared with 25 LMH (without surface treatment) with an almost-stable salt rejection of 87%.

### 3.2. Effect of Different Irradiations on Chemically Modified Membrane 3TG

**Figure 6:**
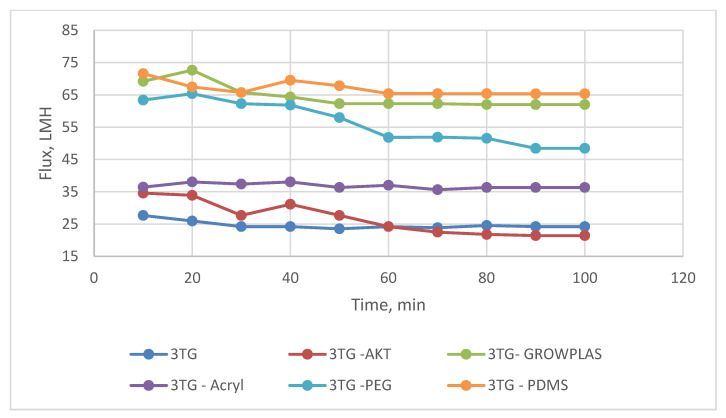
3TG Flux variation with permeation time for different irradiations.

**Figure 7:**
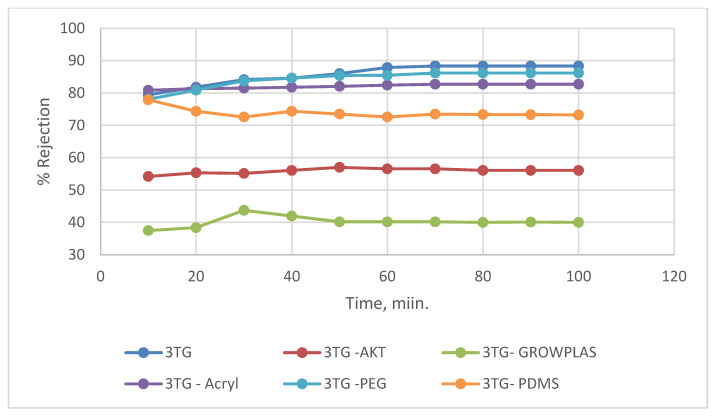
3TG % rejection variation with permeation time for different irradiations.

The change in the chemical structure of the membrane’s top surface was found to greatly affect the efficiency of the applied surface irradiation. The 3TG membranes were prepared from 3T with the grafting and formation of polymethacrylic acid (PMAA). The effects of the different plasmas and VUV irradiation on the membrane surface changed, and their performances were evaluated and presented in Figure 6 and Figure 7. It was found that flux declined to 21 LMH for 3TG with VUV activation (AKT), which is less than the permeate flux for 3TG, and this can be attributed to more chemical interactions in the polymethacrylic layer, penetrating the TFC layer, which suddenly decreased the rejection to about 50%. The percentage rejection (Figure 7) of VUV-PEG shows a slight decrease in rejection of about 86% when compared with the 3TG without irradiation percentage of about 88%. However, it can be assumed to be the best treatment for 3TG, as the permeate flux was raised from 21 LMH (3TG) to 51.5 LMH(VUV-PEG) to almost two and half the productivity with a rejection of 86%.

**Figure 8:**
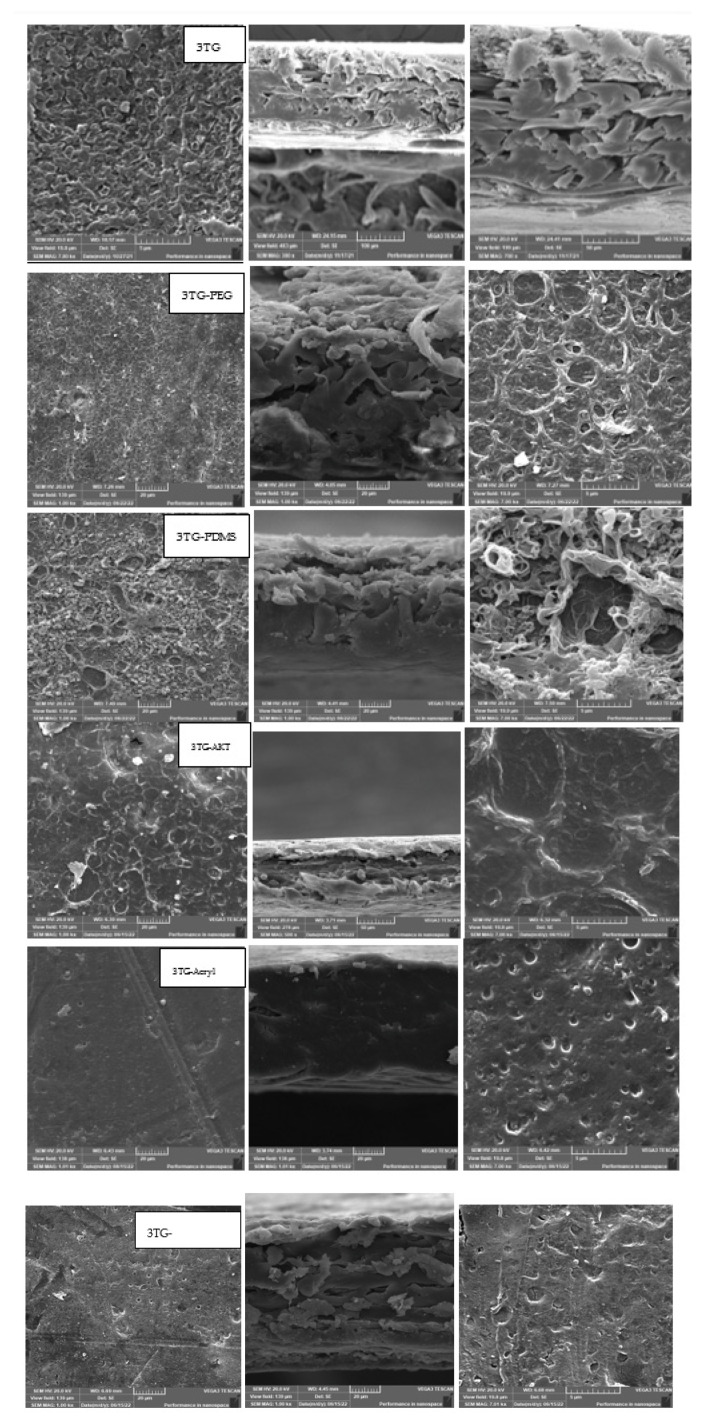
SEM morphology for 3TG with different irradiations.

This means that irradiation efficiency is highly affected by the chemical structure of the beneath layer and upper layer, as the optimum radiation regime changes between 3T and 3TG to be acryl and VUV-PEG, respectively. The sample 3TG-Acryl showed a percentage rejection of about 82%, and another decrease was found in the case of samples 3TG-AKT and 3TG-GrowPLAS to reach 56% and 40%, respectively, as shown in Figure 7. This shows that the presence of these coatings on the polyamide layer does not protect against its deterioration while being subjected to GrowPLAS, which is the worst. Again, activation with VUV showed slightly better but still insufficient results. Coatings with plasma polymeric acrylic acid were more efficient. From Figure 8, one can notice easily that the dense cross-sectional layer found for 3TG-Acryl decreased the flux to 35 LMH with a closed droplet structure in the top surface, but the rejection decreased to 82%. This can be attributed to some slight increase in the pore structure.

The permeate flux shown in Figure 6 has shown that the maximum value of 65 LMH was reached for both 3TG-PDMS and decreased to reach 62 LMH for 3TG-GrowPLAS and about 52.5 LMH for 3TG-PEG. From Figure 8, SEM morphologies can help in the interpretation of such findings, as PDMS and PEG show a similarity in their top structures of open circles when compared to the closed, dense structure in 3TG-Acryl. On the other hand, for 3TG-GrowPLAS, the increase in flux was coupled with the sharp decline in rejection percentage. This can be easily explained by the deep cracks found on its top surface, denoting the etching of the PA layer (Figure 6). 

The effect on different surface irradiations with 3T and 3TG was better explained in Table 2 and Table 3 with XPS data. The oxygen percentage in 3T and 3TG without irradiation is found to be 24 and 16%. These percentages increase upon different irradiations, as 3T with acryl reaches oxygen percent (24%) as that of 3TG with the lowest nitrogen percent go in conformity with the mentioned flux and rejections. This can be illustrated more with a glance at the values of nitrogen percentages for AKT, PDMS, PEG, GrowPLAS, and acryl, which are minimum for acryl and PDMS. However, the optimum is the treatment with the lowest nitrogen content, which is the plasma polymeric acrylic acid coating. The low nitrogen content is assigned to full coverage of the polyamide layer by the plasma polymeric acrylic acid coating. For VUV activation (AKT), it was found that the oxygen percent elevated compared with irradiated 3T by incorporation of oxygen into the surface and formation of carbonyl groups.

### 3.3. Effect of Different Irradiation on Blend Membranes with PVA-3P

The blend membranes without TFC did not obtain as much high salt rejection, but they are representing a facile way to have a comparison tool of interaction between certain irradiation methods and the membrane top surface. The effect of different irradiation techniques on the blended substrate with a PVA cross-linked active layer was studied by recording the percentage rejection of synthetic salt solution (NaCl and CaCl_2_). Knowing that, the rejection of these membranes was low compared with 3T and 3TG but was studied as low-cost and low-pressure (10 bar) RO membranes for desalination with a maximum rejection percentage of about 66% to be applied in certain pretreatment actions.

This active layer which achieves such salt rejection was attributed to the presence of nanographene oxide in the polymeric substrate and crosslinked PVA/GA layer.

**Figure 9:**
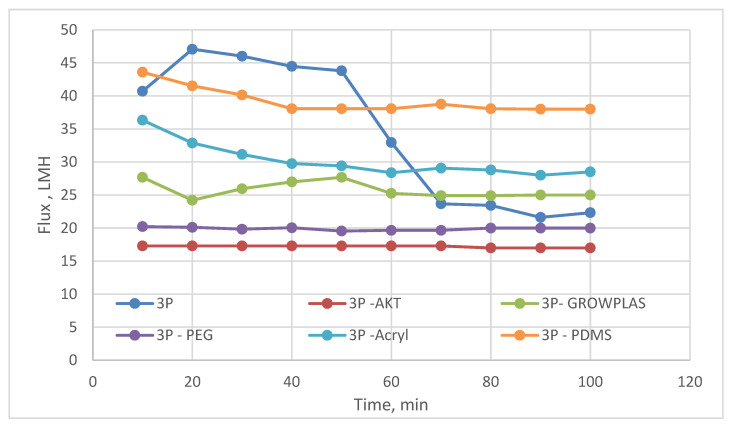
3P Flux variation with permeation time for different irradiations.

By comparing the permeate flux of 3P while being surface modified with different VUVs and plasmas, it was clear that 3P (without irradiation) shows a sharp decline in flux with time due to its high fouling behavior. This may be attributed to the presence of nonhomogeneity in the chemical deposition. A change in permeate flux was found for different irradiations; this shows an enhanced effect when compared to the unmodified membranes (Figure 9).

**Figure 10:**
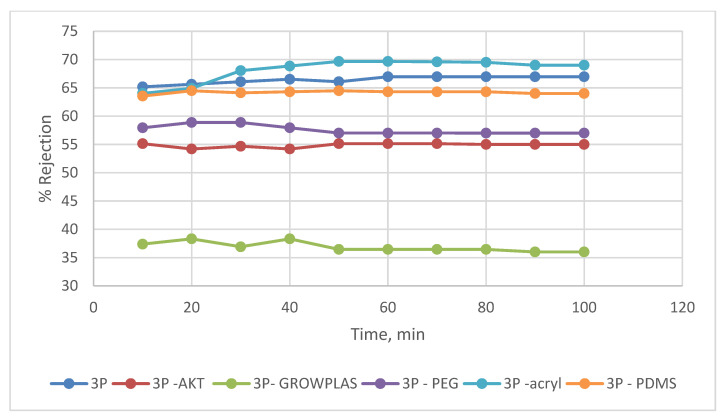
3P % rejection variation with permeation time for different irradiations.

**Figure 11:**
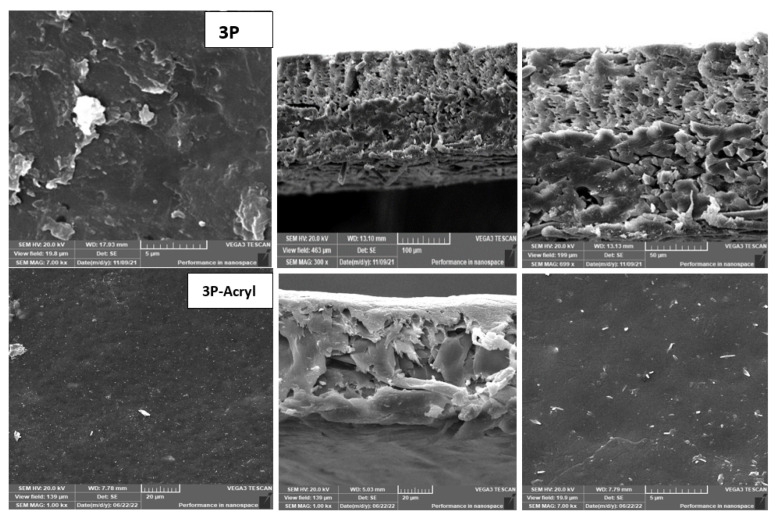
SEM morphology for 3P with and without plasma polymeric acrylic acid coating.

However, a better compromise can be made between the increase in permeate flux with a reasonable salt rejection, as shown in Figure 9 and Figure 10, with 3P-Acryl. It was found that a reasonable increase in salt rejection to 69%, which is clear in the tight, dense structure shown in Figure 11, and with slightly increased permeate water flux from 23 to 29 LMH for 3P and 3P-Acryl, respectively.

The change in the structure of the top surface and cross-sectional plots for the 3P with the best treatment technique (plasma polymeric acrylic acid) compared with the unirradiated 3P are shown in SEM morphology—Figure 11.

The VUV treatment in presence of poly-dimethyl siloxane (PDMS) shows a better influence on 3P when compared to other irradiations with VUV and plasma with a slight decrease in rejection to 64% but an increase in permeate flux to about 38 LMH.

### 3.4. Effect of Physical Irradiation on Grafted MAA-Blend Membranes/PVA

The effect of different plasma and VUV irradiations on double-layered, chemically modified, mixed polymeric substrates will be presented in this section.

**Figure 12:**
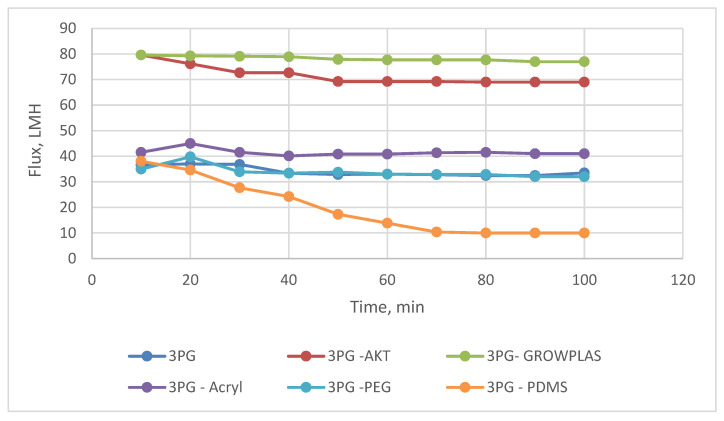
3PG Flux variation with permeation time for different irradiations.

**Figure 13:**
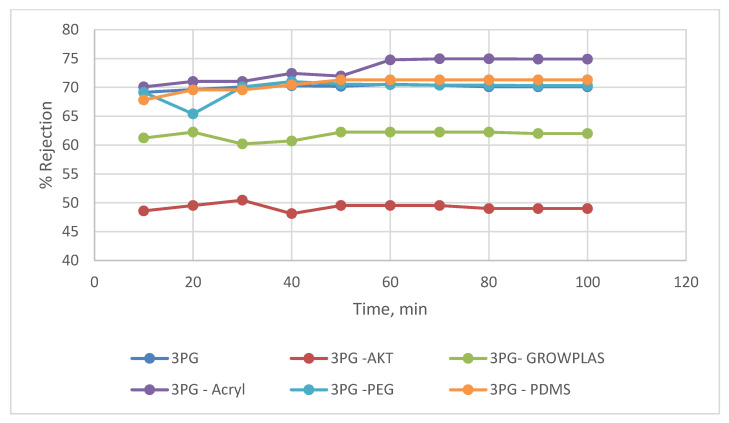
3PG % rejection variation with permeation time for different irradiations.

**Figure 14:**
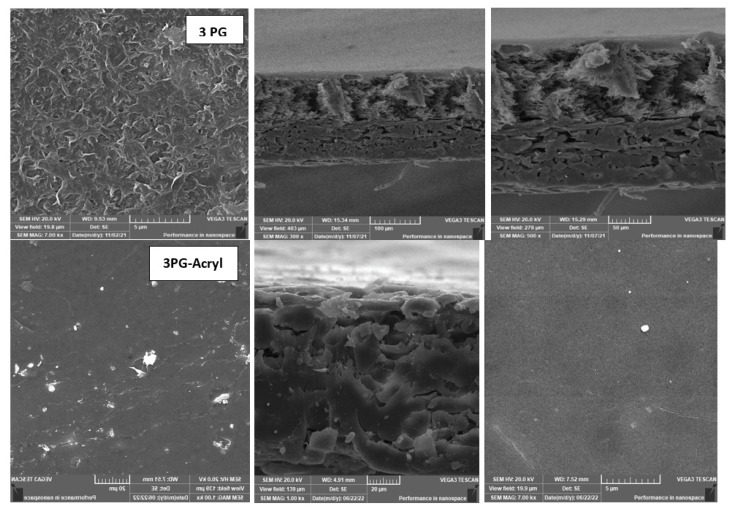
SEM morphology of 3PG membranes with and without plasma irradiation and polyacrylic acid.

The layer-by-layer technique on the membrane surface was studied to highlight the effect of each treatment on salt rejection and permeate flux at a constant feed pressure (20 bar), as shown in Figure 12 and Figure 13. The presence of grafted blend membranes [19] was used as the base structure of different physical irradiations of plasma and VUV. Figure 14 shows the change in morphology recorded by SEM while comparing the top layer of 3PG and the best physical surface modification by means of plasma treatment using polyacrylic acid. This was presented as 3PG-Acryl, which is the best in terms of salt rejection and permeate flux of about 74% and 40 LMH.

This study was important to show the effect of surface irradiation on the presence of a hydrophilic layer of PVA on blended structures (PG family).

This shows a relative enhancement when compared with the salt rejection of 71% and flux of 32 LMH for nonmodified 3PG. The increase in flux for 3PG-GrowPLAS (plasma) and 3PG-AKT(VUV) was attributed to structural failures in the selective layer.

The change in cross-section morphology with the top surface shown in Figure 14 can give a better interpretation of such achievements due to the deposition of the dense uniform layer with increased tiny pores and the deposited polymeric agglomerations spread over the surface of 3PG-Acryl.

### 3.5. Effect of Physical Irradiation on Blank Polymeric Support

**Figure 15:**
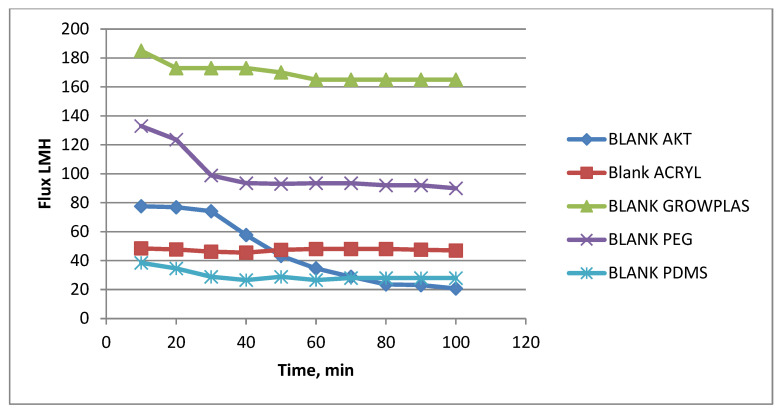
Blank Flux variation with permeation time for different irradiations.

**Figure 16:**
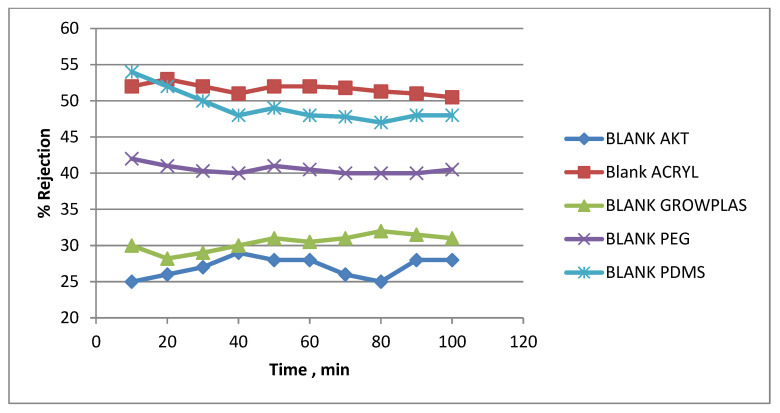
Blank % rejection variation with permeation time for different irradiations.

The effect of different irradiations on the blank-blend polymeric substrate (support) composed of PSF, PAN, and GO dissolved in DMAc will be presented in this section. This was studied to highlight the effect of each treatment technique on prepared membrane behavior in terms of permeate flux, % rejection, antifouling property, flux decline, and finally, chlorine resistance. Blank samples treated by different irradiations show slight differences to the naked eye, as seen in Figure 15 and Figure 16 in the example with VUV-PEG.

**Figure 17:**
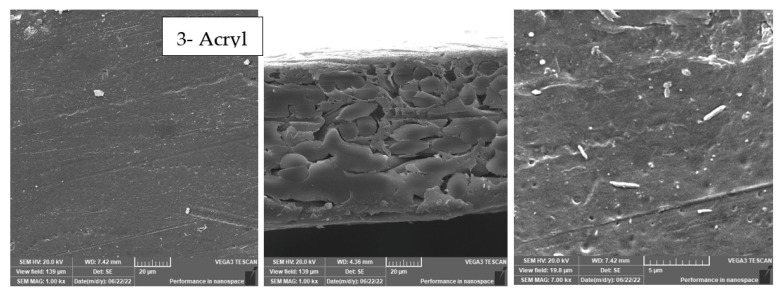
SEM morphology for blank membrane with plasma irradiation (polyacrylic acid).

These membranes have neither a TFC layer nor a PVA layer. Therefore, any attained percentage rejection will be gained by physical surface modification with plasma or VUV. From Figure 15, one can notice easily that the treatment with plasma using GrowPLAS or polyacrylic acid (acryl) shows nearly stabilized permeate flux with the increase in permeation time. The same can be repeated for VUV-polydimethyl siloxane (PDMS), but the target here is well-known to be both higher flux, stability, and salt rejection percentage. For acryl, salt % rejection was found to reach about 50% at a stabilized permeate flux of 48 LMH, which was tripled to reach 165 LMH for GrowPLAS, but the rejection % decreased sharply to nearly 30% of the untreated blank in Figure 16. The plot of the SEM morphology (Figure 17) for the blank-acryl shows a tight, dense structure with the existence of pores, which is responsible for the increase in % rejection with a slight increase from 20 to 28 LMH.

These results can be easily explained, as shown in the following tables. Table 4 and Table 5 show the elemental analysis for the top surfaces of the blank membrane samples as treated with irradiation for surface modification.

Here, we can notice that irradiation using the plasma treatment for Grow Plasma (GrowPLAS) or polyacrylic acid (ACRYL) greatly changes the % C in ACRYL at about 74%, corresponding to higher -COOH groups, which enhance the performance of the membranes.

**Figure 18:**
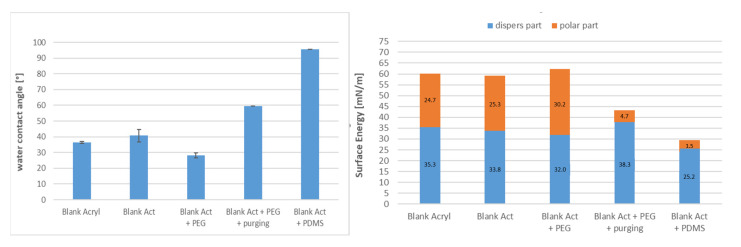
Contact angle and surface energy for blank samples with different irradiations.

By measuring the contact angle (Figure 18), the best treatment to decrease the contact angle and enhance hydrophilicity and antifouling was found: VUV-PEG, which has 29 °C. On the other hand, this treatment increases the surface energy to a maximum of 60 mN/m, divided into nearly equal halves for dispersed and polar parts.

According to the above reached results and optimizations, a great effect for different irradiations was found for 3T and 3TG, which was more than for 3P and 3PG and shows a lot of deformations except for the acryl plasma treatment. So, the antifouling behavior and chlorine resistance were studied for 3T, 3TG, and the blank ones.

### 3.6. Antifouling Behavior for 3T, 3TG, and Blank Membranes

**Figure 19:**
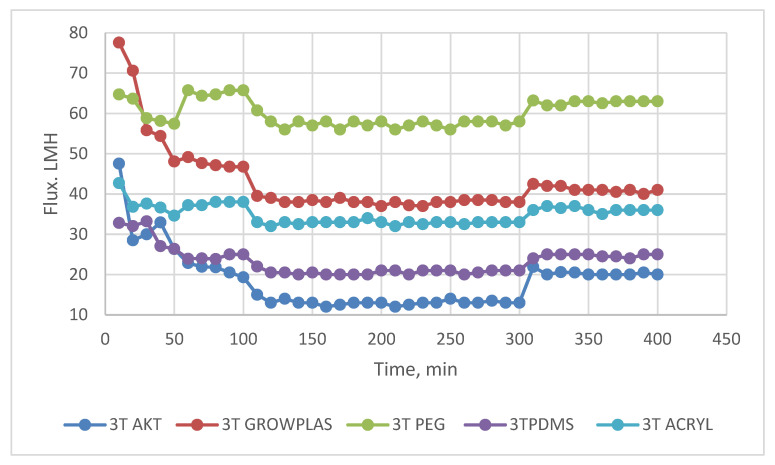
3T flux decline with permeation time for different fouling media.

**Figure 20:**
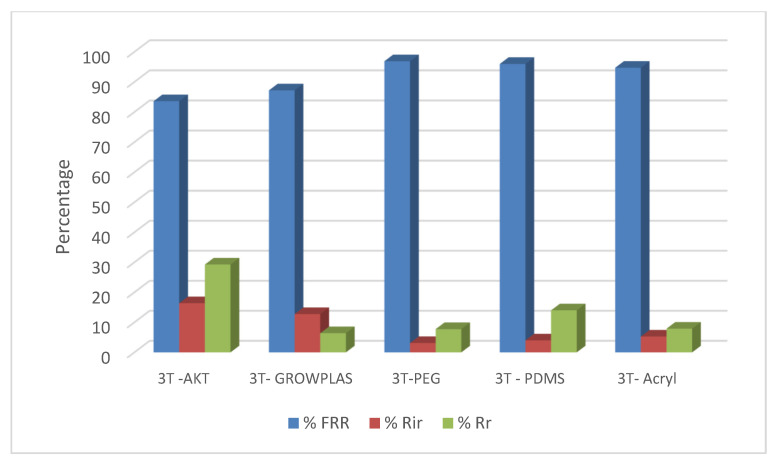
3T family and effect of Irradiations on fouling indicators.

**Figure 21:**
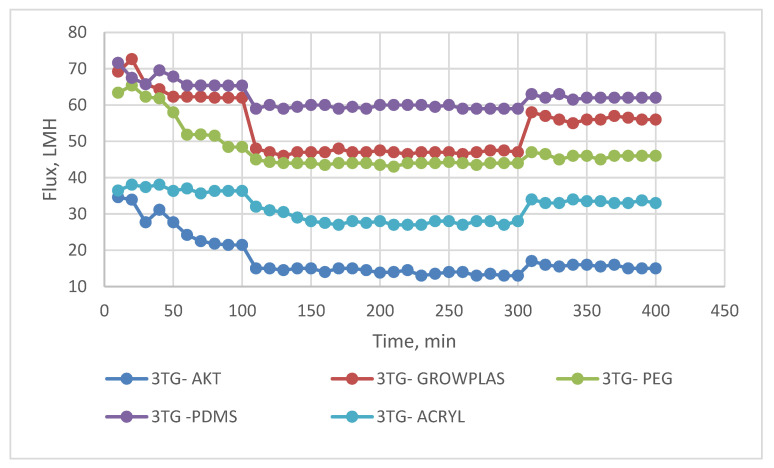
3TG membrane flux with permeation time with foulant solution.

**Figure 22:**
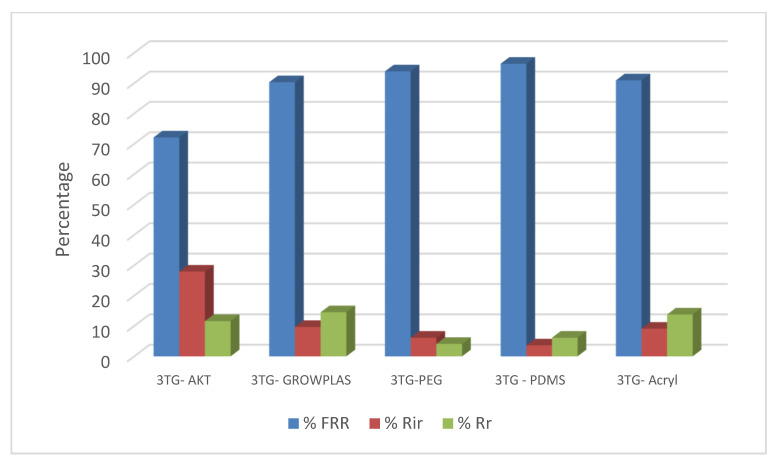
3TG membrane and effect of irradiations on fouling indicators.

**Figure 23:**
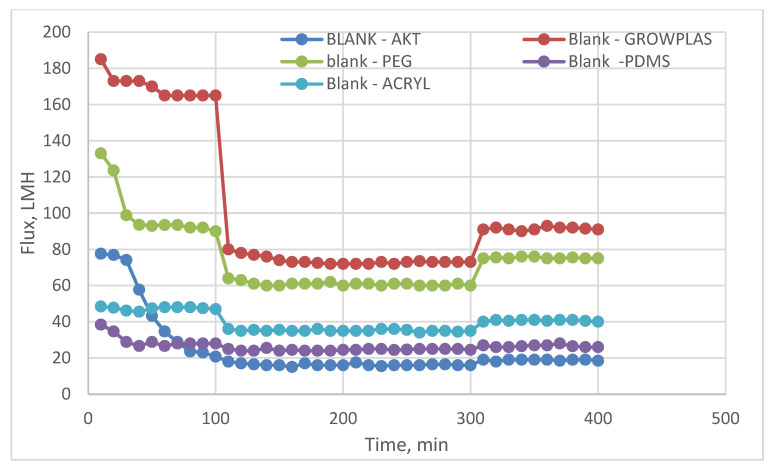
Blank flux decline with permeation time for different fouling media.

**Figure 24:**
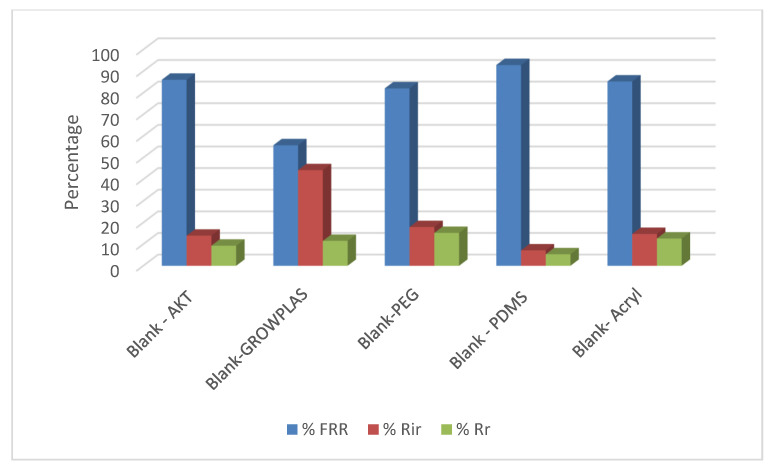
3TG family and effect of irradiations on fouling indicators.

The following experiment recording flux decline and fouling indicators for 3T represented in Figure 19 and Figure 20, for 3TG in Figure 21 and Figure 22, and finally for blank membranes at Figure 23 and Figure 24. It was based upon a study on fouling through permeate flux recordings through three stages. The first was presented by taking synthetic feed solution (NaCl and CaCl_2_) filtration for 100 min followed by a mixed foulant solution (Bovine Serum BSA and Sodium Alginate SA) for 200 min. Then, the membrane was removed from the system and rinsed with distilled water. The flux recovery ratio, reversible and irreversible fouling for 3T, 3TG and blank in Figure 20, Figure 22 and Figure 24 respectively. The feed solution was filtered again and the corresponding flux was recorded, as shown in Figure 19 for 3T, Figure 21 for 3TG, and Figure 23 for the blank membranes.

The fouling indicators showing the flux recovery ratio and the reversible and irreversible fouling are presented for 3T, 3TG, and the blank membranes in Figure 20, Figure 22 and Figure 24, respectively.

For 3T, the maximum % FRR was found for VUV-PEG (96%), and so the minimum irreversible fouling was 3%.

For 3TG, the maximum % FRR was about 96% for VUV-PDMS while the minimum % FRR for 3T and 3TG were 83% and 72%, respectively, for VUV activation (AKT).

In the case of the blank membranes without TFC, the best treatment with respect to antifouling behavior was VUV-PDMS, which obtained the highest % FRR of about 92% while the worst treatment, which decreased % FRR, was the plasma treatment—GrowPLAS, which obtained a percentage of 55.

### 3.7. Effect of Chlorination on Different Surface Irradiations

The effect of different irradiations was determined and compared with previous studies on the effect of GO nanostructures on RO chlorine resistance, and antifouling was studied by the authors in [20], but in a different pattern, in addition to exploring blend membrane efficiencies by nanostructures [21].

1. Effect of Chlorination on VUV Activation (AKT) for different membranes 3T, 3TG, and Blank

The effect of VUV activation (AKT) was studied over different membranes after taking a very high shock of chlorine dose (1000 ppm) to show the layer efficiency to withstand.

**Figure 25:**
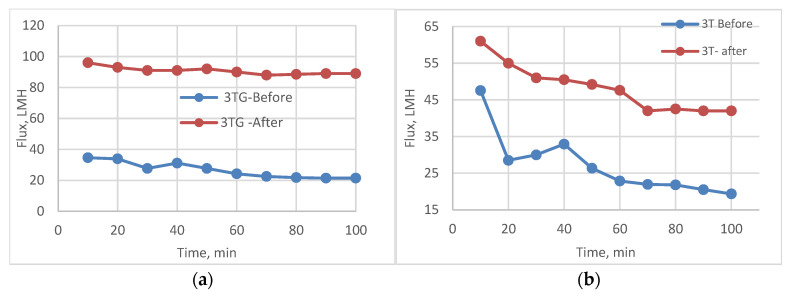

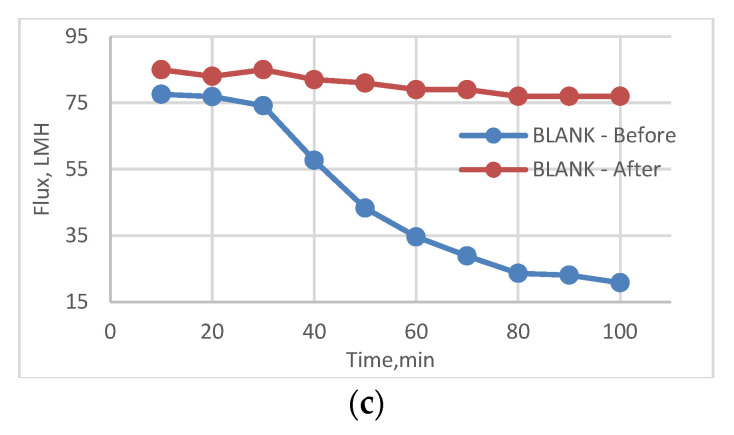
Variation of flux before and after chlorination for VUV-treated membranes(AKT) (**a**) 3TG, (**b**) 3T, and (**c**) blank membranes.

In Figure 25, the change in flux is highest in the 3TG samples (80 LMH) while the 3T and blank samples were lowest at 20 and 60 LMH, respectively. This can be explained by the structure of the polyamide layer interaction with the activation done by the VUV lamps. Therefore, VUV radiation attacks the polyamide layer and accelerates the deterioration of the TFC layer with chlorine as shown with rejection percentage in Table 6.

2. Effect of Chlorination on Plasma treatment with GrowPLAS for different membranes 3T, 3TG, and Blank

**Figure 26:**
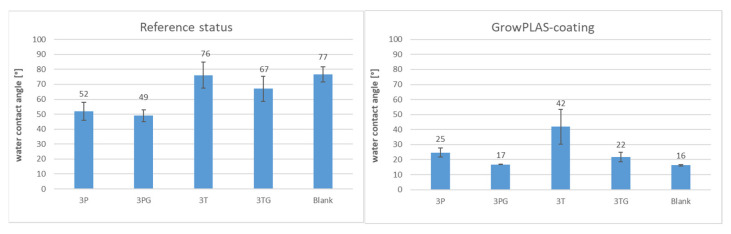
Contact angle comparison before and after GrowPLAS treatment.

**Figure 27:**
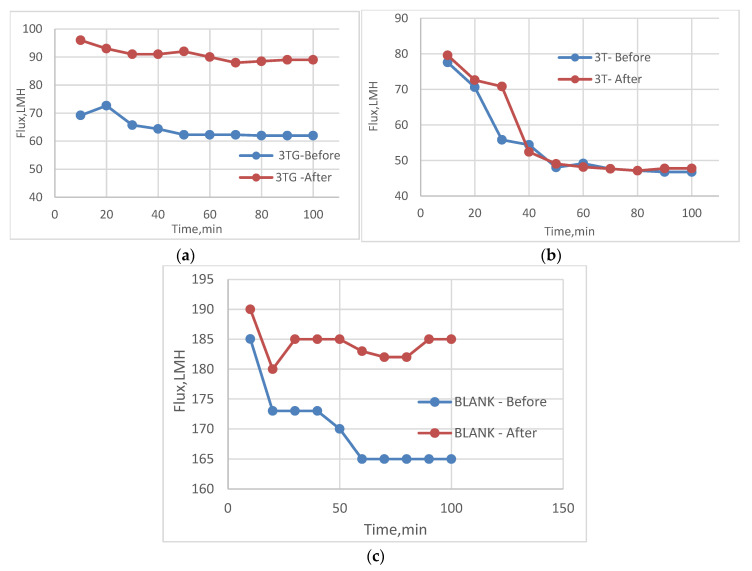
The change in flux variation before and after chlorination with GrowPLAS treatment for 3TG (**a**), 3T (**b**), and blank (**c**).

The effect of GrowPLAS treatment on 3T, 3TG, and blank membranes is shown in the following paragraphs. The contact angles before and after treatments were highly changed, showing a decrease to 22 °C for 3TG, which was 67 °C before GrowPLAS (Figure 26). But for 3T, the contact angle also decreased to reach 42 °C but not similar to 3TG. For chlorine resistance, 3T shows better chlorine resistance, as the flux seems to be unchanged, reflecting the perfect resistance layer, as shown in Figure 27c and so for rejection change shown in Table 7.

**Figure 28:**
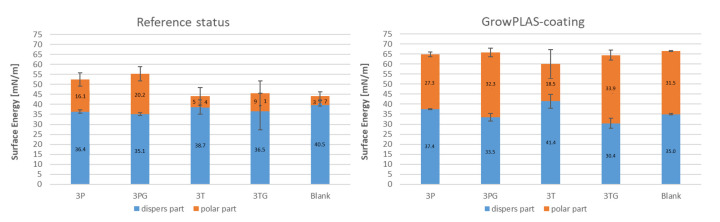
Surface energy variation before and after GrowPLAS treatment.

The effect of GrowPLAS on surface energy is presented in Figure 28. It was clear that the polarity increased for 3T, as the highest membrane was 18.5 mN/m from 5.4 mN/m, and this may increase its superior achieved properties.

3. Effect of Chlorination on Plasma treatment with Polyacrylic acid (Acryl) for different membranes 3T, 3TG, and Blank

The effect of chlorination on the membranes treated with plasma in the presence of polyacrylic acid and the polymerization reaction over the membrane surface produce a homogeneous modification, as shown before in the enhanced flux and percentage rejection (Table 8).

**Figure 29:**
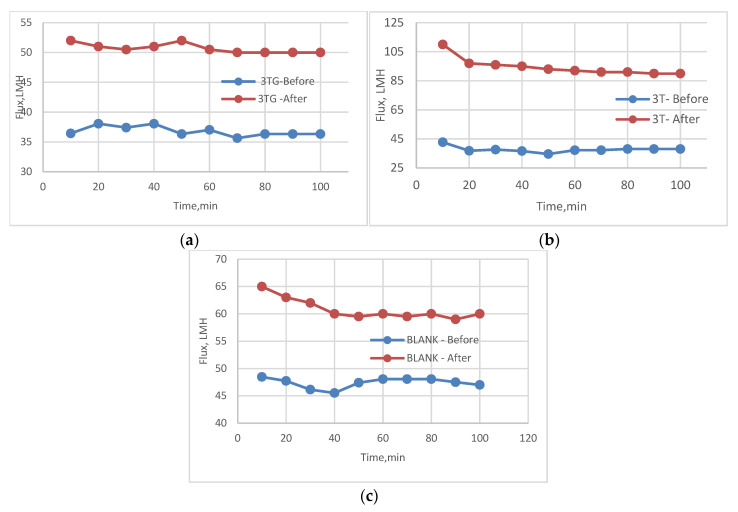
The change in flux variation before and after chlorination with plasma treatment (polyacrylic acid (acryl)) for 3TG (**a**), 3T (**b**), and blank (**c**).

The chlorination with such a high and sudden dose reflects a hard model for such a study. The best chlorine resistance layer for polyacrylic acid applied by plasma was for 3TG, where the rate of change in flux before and after was the least when compared to blank membranes, which came after, and the worst was 3T, as shown in Figure 29. This means that the interaction between layers plays an important role in the behavior of such surface modifications.

4. Effect of Chlorination on VUV with Polyethylene glycol (PEG) for different membranes 3T, 3TG, and Blank

**Figure 30:**
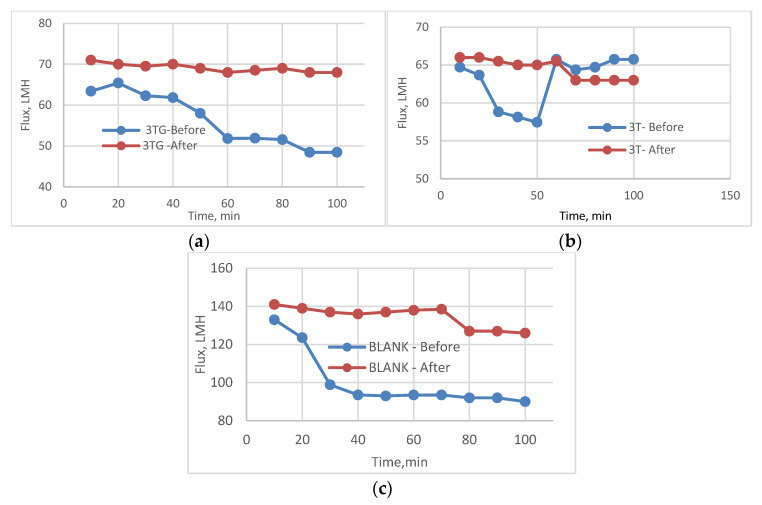
The change in flux variation before and after chlorination with VUV-PEG treatment for 3TG (**a**), 3T (**b**), and blank (**c**).

In Figure 30a–c, the comparison between the behavior of 3T, 3TG, and the blank membranes with different polymeric surface structures used with VUV-PEG. The first with PEG is 3T, which acts to have a defending layer that stops chlorine attack where the flux change is almost zero. After, 3TG also shows a better chlorine resistance compared to the blank samples and this can be confirmed with rejection percentage change in Table 9.

5. Effect of Chlorination on VUV with Poly Di-Methyl Siloxane (PDMS) for different membranes 3T, 3TG, and Blank

**Figure 31:**
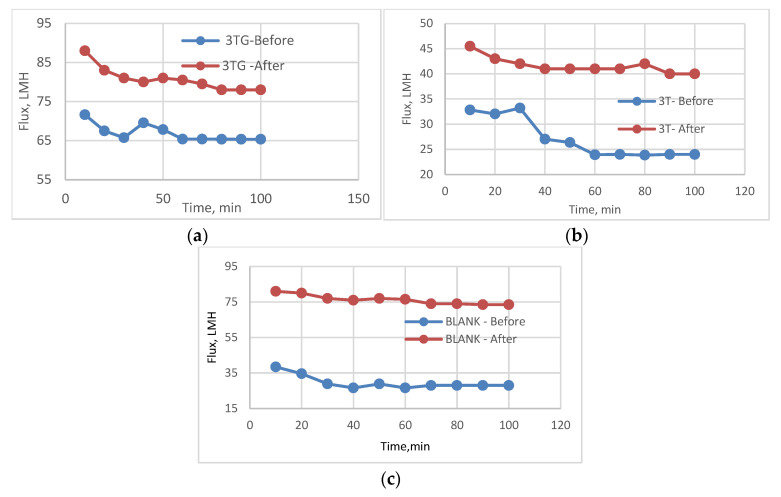
The change in flux variation before and after chlorination with VUV-PDMS treatment for 3TG (**a**), 3T (**b**), and blank (**c**).

The effect of PDMS grafting through VUV irradiation on membranes is clearly presented in Figure 31 and Table 8 before and after chlorination. This irradiation was highly dependent on the top layer, and this was clear in the variation in flux and rejection of the same surface modification with the changed support structure. This can be illustrated more with the blank support, which shows some deformation in the active layer for support. This led to an increase in flux of 40% with a sharp decrease in performance; however, VUV-PDMS has an adverse effect on the poly-methacrylic acid layer from dimethyl-siloxane and was found to decrease the rejection percentage to 73% and 70% before and after chlorination (Table 10).

This can be attributed to different interactions between different treatments and the chemical composition of the top layer.

## 4. Conclusions

Antichlorine, antifouling PSU-PAN/GO membranes were prepared by immersion precipitation and have a TFC layer and were then modified by plasma or vacuum UV irradiation in the presence (3TG) and absence (3T) of a grafted layer with poly-methacrylic acid to enhance the fouling-resistance and shock doses for chlorine exposure of the membrane surface. The effect of different plasma and vacuum UV irradiations was presented on blend polymeric substrates to nominated 3P and 3PG together with the blank blend without any chemical treatments. Vacuum UV and plasma irradiations were introduced, and membrane morphology, performance, and membrane structure were elucidated. Although, the achievement of this work depends on the salt rejection percentages in the range of 88–90%, and this sounds strange for RO rejections. However, these values are comparable with those reached with commercial, RO brackish membranes, which were subjected to long-term, low-pressure testing procedures and found to be 91%.

The following points have been outlined in this study:

1. The mixed-polymeric substrate based upon PSU/PAN/GO showed a great influence on RO membrane surface hydrophilicity, which was greatly affected by the deposition of a plasma polymeric acrylic acid coating (acryl).
2. The enhancement in membrane performance by the plasma polymeric acrylic acid coating in terms of permeate flux and salt rejection was measured at a constant pressure of 20 bar and was proven for blank samples.
3. Additional surface treatments of 3TG membranes by VUV and plasma processes showed a large change in performance. The best was 3TG-PEG, and this confirms the interaction of the top layer with radiation.
4. The achieved performance was confirmed by XPS, SEM, and contact angle analyses, which also confirmed chemical and morphological changes on the membrane surface before and after modification.
5. Antifouling behavior was improved for 3T-PEG, where % FRR was found to be 96% and so minimum irreversible fouling was 3%.
6. In the case of the 3TG and blank membranes, the best % FRR was achieved with VUV-PDMS to obtain 96% and 92% respectively. This means that VUV treatment results in good antifouling effects.
7. Chlorine resistance was enhanced and presented with changes in flux and salt rejection before and after chlorine exposure with high doses. It was found that each membrane showed some better behavior with VUV polymeric grafting with PEG and with polymeric plasma irradiation with either acryl or grow plasma, but for VUV activation, it was the worst.

The achieved modification can be applied easily to produce cost-effective, low-pressure RO membranes with chlorine resistance and antifouling behavior and can be applied to different multiple-stage RO brackish water desalination plants. These surface modifications were assumed to increase membrane lifetime, and so membrane operational and maintenance costs (OPEX) can decrease, this in terms of decreasing chemical cleaning agents and their environmental pollution load.

## Figures and Tables

**Table 1:** Map of physical irradiation over optimized chemically modified membranes.

Membrane	Physical Surface Modification (Irradiation)				
	Low-Pressure Plasma		Vacuum Ultraviolet (VUV)		
	Low-Pressure Plasma—GrowPLAS	Low-Pressure Plasma (Polyacrylic Acid)—Acryl	Vacuum UV (Activation)—AKT	Vacuum UV (Polyethylene Glycol)—PEG	Vacuum UV (Poly-SiOx)—PDMS
3T	GrowPLAS over polyamide layer	Acryl-PLAS over polyamide layer	VUV-AKT over polyamide layer	VUV-PEG over polyamide layer	VUV-PDMS over polyamide layer
3TG	GrowPLAS over polyamide/grafted PMAA	Acryl-PLAS over polyamide/grafted PMAA	VUV-AKT over polyamide/grafted PMAA	VUV-PEG over polyamide/grafted PMAA	VUV-PDMS over polyamide/grafted PMAA
3P	GrowPLAS over PVA/GA layer	Acryl-PLAS over PVA/GA layer	VUV-AKT over PVA/GA layer	VUV-PEG over PVA/GA layer	VUV-PDMS over PVA/GA layer
3PG	GrowPLAS over PVA/GA and PMAA	Acryl-PLAS over PVA/GA and PMAA	VUV-AKT over PVA/GA and PMAA	VUV-PEG over PVA/GA and PMAA	VUV-PDMS over PVA/GA and PMAA
Blank	GrowPLAS alone	Acryl-PLAS alone	VUV-AKT alone	VUV-PEG alone	VUV-PDMS alone

**Table 2:** XPS data for 3T after surface irradiation in two positions.

	C (at%)	O (at%)	N (at%)	Si (at%)	Mg (at%)	Cl (at%)	Al (at%)	Na (at%)	Ca (at%)	S (at%)
3TG Ref, Pos1	66.3	24.1	4.0	0.3	0.4	0.9	0.2	0.8	2.6	0.4
3TG Ref, Pos2	64.5	25.8	3.0	1.1	0.3	0.8	0.9	0.7	2.7	0.3
3T Ref, Pos1	72.6	16.0	9.9	0.4	-	0.8	-	0.2	0.2	<0.1
3T Ref, Pos2	71.9	16.4	10.0	0.4	-	0.8	-	0.2	0.2	<0.1
Akt, Pos1	63.3	23.6	11.1	<0.1	0.2	0.7	-	0.5	0.5	<0.1
Akt, Pos2	62.6	24.0	11.0	0.1	0.2	0.8	-	0.5	0.8	<0.1
Grow, Pos1	42.8	35.5	7.1	13.7	-	0.3	-	<0.1	0.1	0.4
Grow, Pos2	47.8	31.2	8.5	11.8	-	0.3	-	<0.1	0.2	0.2
PDMS, Pos1	16.4	57.6	2.6	23.2	-	<0.1	-	-	<0.1	<0.1
PDMS, Pos2	10.4	60.8	1.1	27.8	-	-	-	-	-	-
PEG, Pos1	60.9	26.7	9.8	0.4	0.4	0.6	-	0.3	0.9	0.2
PEG, Pos2	61.8	26.7	9.4	0.5	0.2	0.4	-	0.3	0.5	0.3
Acryl, Pos1	73.5	25.2	0.4	-	-	-	-	1.0	-	-
Acryl, Pos2	74.8	24.0	0.3	-	-	-	-	0.9	-	-

**Table 3:** Functional groups formed after treatment.

	C-C/C-H (%C)	-C-OH/C-O-C or –C-NH (%C)	C=O (%C)	-COOR/-COOH (%C)	π-π* Shake Up (%C)
3TG Ref, Pos1	67.0	16.4	13.7	2.8	-
3TG Ref, Pos2	69.9	14.9	12.8	2.2	0.2
3T Ref, Pos1	70.5	15.5	12.2	0.2	1.5
3T Ref, Pos2	69.5	16.2	10.9	1.3	2.1
Akt, Pos1	60.5	11.9	23.2	0.4	4.1
Akt, Pos2	61.4	11.2	23.1	1.1	3.3
Grow, Pos1	72.1	19.0	6.4	1.2	1.4
Grow, Pos2	72.3	20.7	4.9	1.6	0.6
PDMS, Pos1	62.2	13.5	17.8	5.5	1.0
PDMS, Pos2	62.7	16.3	12.4	7.6	1.1
PEG, Pos1	52.6	21.8	19.7	-	3.8
PEG, Pos2	49.7	19.5	18.0	1.2	3.8
Acryl, Pos1	67.9	14.3	6.9	11.0	-
Acryl, Pos2	69.7	13.6	6.8	9.7	0.2

**Table 4:** XPS measurements for blank samples with different irradiations and elemental analysis.

Treatment	C (at%)	O (at%)	Cl (at%)	N (at%)	Si (at%)	Al (at%)	S (at%)	Ca (at%)	Mg (at%)	Na (at%)
Blank Reference	76.0	16.5	0.15	2.5	1.4	0.25	2.6	0.25	0.15	-
Blank GrowPLAS	30.4	49.5	-	1.6	16.7	0.45	1.1	0.2	-	0.15
Blank Acryl	74.2	25.4	-	0.35	-	-	-	-	-	-
Blank Activation	58.0	30.3	0.1	4.9	1.9	0.55	2.7	0.3	0.3	0.95
Blank Act + PEG	66.1	26.9	0.4	1.7	1.0	0.35	1.4	0.6	0.55	1.0
Blank Act + PEG + purging	77.8	18.5	0.1	1.7	0.85	0.2	0.55	0.1	0.1	0.25
Blank Act + PDMS	11.3	60.1	-	0.8	27.2	-	0.65	-	-	0.1
Wafer Act	5.0	35.9	-	-	59.1	-	-	-	-	0.1
Wafer Act + PEG	84.7	13.7	-	1.0	0.5	-	0.1	-	-	0.25
Wafer Act + PEG + purging	70.2	21.6	-	1.0	7.3	-	-	0.1	-	0.1
Wafer Act + PDMS	10.5	60.7	-	0.2	28.8	-	-	-	-	-

**Table 5:** XPS measurements for blank samples with different irradiations and functional groups.

Treatment	C-C/C-H (%C)	-C-OH/C-O-C or –C-NH (%C)	C=O (%C)	-COOR/-COOH (%C)	π-π* Shake-Up (%C)
Blank Reference	74.3	21.1	0.9	0.2	3.7
Blank GrowPLAS	56.9	28.0	8.2	6.2	0.75
Blank Acryl	68.4	14.0	5.3	12.5	-
Blank Activation	50.9	32.0	6.2	9.1	2.0
Blank Act + PEG	55.5	33.9	8.3	2.3	0.3
Blank Act + PEG + purging	69.2	22.6	6.9	1.0	0.4
Blank Act + PDMS	67.0	19.7	6.5	6.0	0.9

**Table 6:** Salt rejection variation for membranes treated with VUV activation (AKT) before and after chlorination.

Membrane	% Salt Rejection before Chlorination	% Salt Rejection after Chlorination
3T	71.36	68
3TG	56.54	50.13
Blank”3”	28	25

**Table 7:** Salt rejection variation for membranes treated with grow plasma (GrowPLAS) before and after chlorination.

Membrane	% Salt Rejection before Chlorination	% Salt Rejection after Chlorination
3T	75.7	74.9
3TG	40.17	35.72
Blank”3”	31	28.56

**Table 8:** Salt rejection variation for membranes treated with acryl-plasma (acryl) before and after chlorination.

Membrane	% Salt Rejection before Chlorination	% Salt Rejection after Chlorination
3T	86.86	70.37
3TG	82.68	80
Blank”3”	52	50.8

**Table 9:** Salt rejection variation for membranes treated with VUV-PEG before and after chlorination.

Membrane	% Salt Rejection before Chlorination	% Salt Rejection after Chlorination
3T	75.6	75
3TG	86.17	85.54
Blank”3”	40.5	35

**Table 10:** Salt rejection variation for membranes treated with VUV-PDMS before and after chlorination.

Membrane	% Salt Rejection before Chlorination	% Salt Rejection after Chlorination
3T	84.76	83.2
3TG	73.45	70.72
Blank”3”	48	30

## Data Availability

Not applicable.
